# Identifying disordered eating behaviours in adolescents: how do parent and adolescent reports differ by sex and age?

**DOI:** 10.1007/s00787-016-0935-1

**Published:** 2017-01-03

**Authors:** Savani Bartholdy, Karina Allen, John Hodsoll, Owen G. O’Daly, Iain C. Campbell, Tobias Banaschewski, Arun L. W. Bokde, Uli Bromberg, Christian Büchel, Erin Burke Quinlan, Patricia J. Conrod, Sylvane Desrivières, Herta Flor, Vincent Frouin, Jürgen Gallinat, Hugh Garavan, Andreas Heinz, Bernd Ittermann, Jean-Luc Martinot, Eric Artiges, Frauke Nees, Dimitri Papadopoulos Orfanos, Tomáš Paus, Luise Poustka, Michael N. Smolka, Eva Mennigen, Henrik Walter, Robert Whelan, Gunter Schumann, Ulrike Schmidt

**Affiliations:** 1 Section of Eating Disorders, Department of Psychological Medicine, Institute of Psychiatry, Psychology and Neuroscience, King’s College London, London, UK; 2 South London and Maudsley NHS Foundation Trust, London, UK; 3 Department of Biostatistics, Institute of Psychiatry, Psychology and Neuroscience, King’s College London, London, UK; 4 Centre for Neuroimaging Sciences, Institute of Psychiatry, Psychology and Neuroscience, King’s College London, London, UK; 5 Department of Child and Adolescent Psychiatry and Psychotherapy, Central Institute of Mental Health, Medical Faculty Mannheim, Heidelberg University, Square J5, 68159 Mannheim, Germany; 6 Discipline of Psychiatry, School of Medicine and Trinity College Institute of Neuroscience, Trinity College Dublin, Dublin, Ireland; 7 University Medical Centre Hamburg-Eppendorf, House W34, 3.OG, Martinistr. 52, 20246 Hamburg, Germany; 8 Medical Research Council-Social, Genetic and Developmental Psychiatry Centre, Institute of Psychiatry, Psychology and Neuroscience, King’s College London, London, UK; 9 Department of Psychiatry, Université de Montréal, CHU Ste Justine Hospital, Quebec, Canada; 10 Department of Psychological Medicine and Psychiatry, Institute of Psychiatry, Psychology and Neuroscience, King’s College London, London, UK; 11 Department of Cognitive and Clinical Neuroscience, Central Institute of Mental Health, Medical Faculty Mannheim, Heidelberg University, Square J5, Mannheim, Germany; 12 Neurospin, Commissariat à l’Energie Atomique, CEA-Saclay Center, Paris, France; 13 Department of Psychiatry and Psychotherapy, University Medical Center Hamburg-Eppendorf (UKE), Martinistrasse 52, 20246 Hamburg, Germany; 14 Departments of Psychiatry and Psychology, University of Vermont, Burlington, VT 05405 USA; 15 Department of Psychiatry and Psychotherapy, Campus Charité Mitte, Charité, Universitätsmedizin Berlin, Charitéplatz 1, Berlin, Germany; 16 Physikalisch-Technische Bundesanstalt (PTB), Abbestr. 2-12, Berlin, Germany; 17 Institut National de la Santé et de la Recherche Médicale, INSERM Unit 1000 “Neuroimaging & Psychiatry”, University Paris Sud, University Paris Descartes-Sorbonne Paris Cité, Paris, France; 18 Maison de Solenn, Paris, France; 19 Psychiatry Department 91G16, Orsay Hospital, Orsay, France; 20 Rotman Research Institute, Baycrest and Departments of Psychology and Psychiatry, University of Toronto, Toronto, ON M6A 2E1 Canada; 21 Department of Child and Adolescent Psychiatry and Psychotherapy, Medical University of Vienna, Vienna, Austria; 22 Department of Psychiatry and Neuroimaging Center, Technische Universität Dresden, Dresden, Germany; 23 Department of Psychology, University College Dublin, Dublin, Ireland

**Keywords:** Parent, Adolescent, Epidemiology, Eating disorders

## Abstract

This study investigated the prevalence of disordered eating cognitions and behaviours across mid-adolescence in a large European sample, and explored the extent to which prevalence ratings were affected by informant (parent/adolescent), or the sex or age of the adolescent. The Development and Well-Being Assessment was completed by parent–adolescent dyads at age 14 (*n* = 2225) and again at age 16 (*n* = 1607) to explore the prevalence of 7 eating disorder symptoms (binge eating, purging, fear of weight gain, distress over shape/weight, avoidance of fattening foods, food restriction, and exercise for weight loss). Informant agreement was assessed using kappa coefficients. Generalised estimating equations were performed to explore the impact of age, sex and informant on symptom prevalence. Slight to fair agreement was observed between parent and adolescent reports (kappa estimates between 0.045 and 0.318); however, this was largely driven by agreement on the absence of behaviours. Disordered eating behaviours were more consistently endorsed amongst girls compared to boys (odds ratios: 2.96–5.90) and by adolescents compared to their parents (odds ratios: 2.71–9.05). Our data are consistent with previous findings in epidemiological studies. The findings suggest that sex-related differences in the prevalence of disordered eating behaviour are established by mid-adolescence. The greater prevalence rates obtained from adolescent compared to parent reports may be due to the secretive nature of the behaviours and/or lack of awareness by parents. If adolescent reports are overlooked, the disordered behaviour may have a greater opportunity to become more entrenched.

## Introduction

Eating disorders (EDs) are characterised by pathological concerns over shape and weight, and disturbed eating and weight-control behaviour. They are more common in females and typically start during adolescence, with a peak onset between ages 15 and 20 [1–3]. However, the age at which disordered eating behaviours (DEBs) and associated cognitions initially develop has not been widely studied, and it is unclear at what age the sex differences in the prevalence of DEBs emerge. Large prospective longitudinal cohort studies of community-dwelling adolescents are required to answer such questions, although the optimal method of assessing DEBs in adolescents remains unclear.

The use of multiple informants in assessing emotional and behavioural problems in youth is often advocated [4], as multiple perspectives of a child’s behaviour are likely to enrich assessment, and can be important in diagnosing disorders involving symptom denial [5]. It has been proposed that parental reports may be beneficial for assessing anorexia nervosa (AN) as sufferers themselves often downplay symptoms at the start of the illness [6]. In contrast, parents may be unaware and under-report behaviours characteristic of bulimia nervosa (BN) that are often associated wtih secrecy and shame, such as binge eating and purging [5]. However, agreement between informants tends to be low [4, 7]. Moreover, reliance on multiple informants may be problematic, as others’ responses may be biased by their own attitudes, personality and internal state [8–10].

Poor-to-moderate agreement between youth and parent ratings has been observed for DEB among clinical samples. For example, Mariano et al. [11] found acceptable agreement for the presence of behavioural symptoms (e.g. binge eating, self-induced vomiting, and laxative/diuretic misuse), but poor agreement on frequency of behaviours and experience of disordered eating cognitions, with greater severity reported by young people compared to their parents. Similarly, Salbach-Andrae et al. [12] observed poor concordance between parent and adolescent reports, particularly for internalising behaviours. While several studies have observed good concordance for symptoms of AN [11], one study reported less concerns over weight and restraint in child reports (aged 6–12 years) compared to their parents [6], and another study revealed greater concordance for adolescents with BN compared to those with AN-Restrictive subtype [12]. In contrast, youths suffering from BN have been found to report greater severity of cognitions and frequency of behaviours [11], shape concerns and restraint [6] compared to their parents. Thus, concordance between parent and youth reports in clinical populations may depend on the nature of the behaviour and the stage or severity of illness.

Similar levels of non-concordance have been reported in non-clinical samples. Studies have reported good agreement on the absence of DEBs and modest agreement for the presence of eating disordered cognitions [13], but poor concordance for bulimic symptoms such as binge eating [13, 14]. One study observed that similar prevalences of DEBs were reported by parents and youth, but found high levels of within-dyad disagreement [5]. Thus, parents may not be aware of their children’s engagement in such behaviours [15]. Additionally, parents and children may differ in their understanding of problematic eating behaviours [5]. It is, therefore, important to assess parent–child agreement on both behavioural and cognitive symptoms to understand how best to identify symptoms amongst young people at a high-risk age (early–mid-adolescence) in the community.

Moreover, there has been little research into factors that affect concordance between youth and parental reports. Only one study has explored the degree to which informant (adolescents and their mothers) and sex influence the prevalence of DEBs in a large UK community sample [5]. The present study aims to (a) characterise the point prevalence of DEBs at ages 14 and 16 in a large multinational community sample based on adolescent self-reports and parental reports [IMAGEN cohort; 16], and (b) explore the concordance between parent and youth ratings of DEBs. This study extends the assessment of parent–youth agreement to a large multinational European cohort to explore the generalisability of findings across cultures. We predict that prevalence of DEBs will be higher in later adolescence (at age 16 compared to 14) and in girls at both ages compared to boys. Based on previous studies assessing multiple-informant agreement on DEBs in non-clinical samples, we hypothesise that greater agreement would be observed on disordered eating cognitions (fear of weight gain, distress over shape and weight), and behaviours (avoidance of fattening foods, food restriction, and exercise for weight loss) compared to binge eating and purging, which are additionally predicted to be more frequently endorsed by adolescent self-reports compared to parent reports, given the secretive nature of these behaviours.

## Materials and methods

### Participants

Participants were those taking part in a large multinational cohort study [the IMAGEN study: for further details, see 16]. Participants at age 14 (time point 1; T1) and their parents were recruited from secondary schools in 8 sites across the UK, Ireland, France and Germany. A total of 2225 parent–adolescent dyads completed the Development and Well-Being Assessment (DAWBA) online at T1; however, only 2215 of these pairs had data from the Dieting, Weight and Body Shape section (assessing ED symptoms) from at least 1 informant [43 dyads had data from only 1 informant (21 dyads with adolescent data only)]. 1607 parent–adolescent dyads also completed the DAWBA when the adolescent was aged 16 (time point 2; T2) (including an additional 2 pairs with incomplete baseline data); however, only 1604 pairs had data from the Dieting, Weight and Body Shape section from at least 1 informant (53 dyads with data from only 1 informant [25 dyads with adolescent data only]).

### Measures

#### DAWBA interview

The DAWBA is a semi-structured interview that assesses the presence and frequency of symptoms of a number of psychiatric disorders. A youth and a parent version of the DAWBA were administered. Following on from a previous study exploring EDs in early adolescence using the DAWBA interview [17], parent–adolescent agreement on the presence/absence of seven specific symptoms within the preceding 3 months was assessed: fear of weight gain (question 8), distress about shape/weight (question 11), avoidance of fattening foods (question 26), food restriction [composite measure including skipping meals (question 18a), eating less at meals (question 18b) and fasting (question 18c)], exercising for weight loss (question 18e), binge eating (eating an objectively large amount of food with associated loss of control; questions 15 and 16) and purging (actively getting rid of ingested food by self-induced vomiting or pill use; questions 1c, 18f and 18g).

#### Body mass index

In adolescents, body mass index (BMI: kg/m^2^) is dependent on age and sex [18]. BMI z-scores were calculated based on the Centre for Disease Control and Prevention (CDC) Growth Charts correcting for age (in months) and sex [19] to determine how an individual’s weight-for-height compares to children of the same age and sex using an external reference standard [18]. Following CDC recommendations, the following cutoffs were used: >=95th percentile for obesity, 85th–95th percentile for overweight, and <5th percentile for underweight.

### Procedure

Interview, questionnaire, genetic and neuroimaging data were obtained from adolescents at age 14 (T1), and interview and questionnaire data were obtained online two years later at age 16 (T2). Interview and questionnaire data were collected from the parents at both time points. Only the responses on the Dieting, Weight and Body Shape section of the Development and Well-Being Assessment (DAWBA) interview were used here. The DAWBA interview was administered online, and height and weight were measured in person. Procedures were approved by the local ethics committees at each site, and were conducted in accordance with the Declaration of Helsinki. Written informed consent from the parents and written assent from the children were obtained prior to participation.

### Statistical analysis

The DAWBA assesses presence and severity using an ordinal scale, providing the following options: “no”, “a little”, “a lot” or “tries but not allowed”. Only participants that responded to the screening questions of the DAWBA Dieting, Weight and Body Shape section were included in the analyses. Responses were dichotomised into presence/absence ratings: consistent ratings of “no” on the section’s screening questions (due to the use of skip rules) or a rating of “no” on the specific symptom question(s) were marked as the DEB being absent, and any of the other ratings were marked as the DEB being present. For binge eating and purging behaviours, one question only assessed presence or absence without assessing severity; however, if the presence of either behaviour was indicated, the participant was included in the counts for these behaviours. Hence, some participants may have reported engaging in binge eating or purging behaviour without providing a frequency rating.

The presence/absence ratings were entered into a 2 × 2 contingency table for each DEB at each time point. Agreement between parent and adolescent ratings was assessed using the kappa coefficient. Values for kappa coefficients range between 0 and 1 (≤0 = poor, 0.01–0.20 = slight, 0.21–0.40 = fair, 0.41–0.60 = moderate, 0.61–0.80 = substantial, 0.81–1 = almost perfect [20]). Symptom prevalence was first explored by calculating the percentage of adolescents for whom the symptom was endorsed by adolescent self-report, parent report, or either informant (using the OR rule). Secondly, we explored parental agreement for children who reported any behaviour at both time points, to establish whether parental agreement increased over time with respect to the adolescents who continued to perceive themselves to be symptomatic, and vice versa. Finally, we modelled the main effects of informant (parent/child), sex (boys/girls) and age (14/16) as predictors for each symptom using generalised estimating equations (GEE). GEE was employed over generalised linear mixed models, as we were interested in the impact of informant, sex and age as predictors at the population level, rather than at a subject-specific level (i.e. population-averaged odds rather than subject-specific odds [21]). As this analysis included within-subjects factors, independence could not be assumed, thereby prohibiting simple logistic regressions. GEE models assume that cases are not independent, assume a correlation between errors (the structure of which are specified by a covariance matrix) and do not assume heterogeneity of variance; thus, the GEE approach can be used to analyse non-normal within-subject data [22]. The GEE employed a binary logistic model with a logit link and an exchangeable working correlation matrix. As our analysis included two time points, the exchangeable and first-order autoregressive working correlation matrices would be expected to produce roughly similar results [23]. Previous studies have also reported similar estimates and standard errors using exchangeable, independent and unspecified working correlation matrices on data assessed at two time points [21]. To deal with missing data, multiple imputation based on fully conditional specification was performed to allow data to be missing at random (MAR). Although GEE models are typically taken to allow data to be missing completely at random, Satty et al. [24] show that GEE with multiple imputation can perform well under the assumption of MAR, conditional on the important predictors of missingness being included in the model. Here, age, sex and informant were included as predictors of missingness. All models were run on 100 imputed datasets, and the presented are pooled estimates combined using Rubin’s rules. All analyses were conducted in IBM SPSS Statistics version 21.

## Results

### Descriptive statistics

Demographic information is shown in Table 1. An approximately equal proportion of boys and girls participated at both time points. Most of the parental reports were provided by the mothers, followed by the fathers. The remaining reports were provided by both parents together, or by caregivers or guardians such as step- or foster parents, other relatives or a residential care worker.

**Table 1:** Participant demographics

	T1 (age 14)				T2 (age 16)			
	Girls	Boys	Missing^a^ (*n*)	Total	Girls	Boys	Missing^a^ (*n*)	Total
Total *n*	1124	1072	29	2225	826	769	12	1607
Mean (SD) age (years)	14.55 (0.45)	14.53 (0.47)	108 (46 f)	14.54 (0.46)	16.50 (0.59)	16.48 (0.638)	87 (42 f)	16.49 (0.61)
Mean (SD) BMI (*z*-score adjusted for age and sex)	0.28 (1.008)	0.25 (1.087)	204 (111 f)	0.26 (1.048)	0.16 (1.305)	0.31 (1.435)	444 (195 f)	0.23 (1.367)
Parent type [*n* (%)]^*b*^								
Parent (unspecified)	25 [2.2%]	19 [1.8%]	2 [12.9%]	46 [2.2%]	46 [5.6%]	45 [5.9%]	2 [16.7%]	93 [5.8%]
Mother	881 [78.5%]	817 [76.2%]	17 [54.8%]	1715 [77.1%]	625 [75.7%]	571 [74.3%]	8 [66.7%]	1204 [74.9%]
Father	181 [16.1%]	207 [19.3%]	6 [19.4%]	394 [17.7%]	122 [14.8%]	118 [15.3%]	2 [16.7%]	242 [15.1%]
Both parents	4 [0.4%]	8 [0.7%]	0 [0.0%]	12 [0.5%]	5 [0.6%]	4 [0.5%]	0 [0.0%]	9 [0.6%]
Other caregiver type	17 [1.5%]	10 [0.9%]	0 [0.0%]	27 [1.2%]	6 [0.7%]	5 [0.7%]	0 [0.0%]	11 [0.7%]
Missing	14 [1.2%]	11 [1.0%]	4 [12.9%]	29 [1.3%]	22 [2.7%]	26 [3.4%]	0 [0.0%]	48 [3.0%]
Site [*n*, (%)]								
London	147 [13.1%]	126 [11.8%]	0 [0.0%]	273 [12.3%]	134 [16.2%]	106 [13.8%]	0 [0.0%]	240 [14.9%]
Nottingham	175 [15.6%]	185 [17.3%]	5 [16.1%]	365 [16.4%]	145 [17.6%]	138 [17.9%]	2 [16.7%]	285 [17.7%]
Dublin	102 [9.1%]	118 [11.0%]	21 [67.7%]	241 [10.8%]	85 [10.3%]	96 [12.5%]	10 [83.3%]	191 [11.9%]
Paris	130 [11.6%]	133 [12.4%]	1 [3.2%]	264 [11.9%]	72 [8.7%]	66 [8.6%]	0 [0.0%]	138 [8.6%]
Berlin	144 [12.8%]	128 [11.9%]	2 [6.5%]	274 [12.3%]	80 [9.7%]	56 [7.3%]	0 [0.0%]	136 [8.5%]
Hamburg	145 [12.9%]	121 [11.3%]	0 [0.0%]	265 [11.9%]	107 [13.0%]	99 [12.9%]	0 [0.0%]	206 [12.8%]
Mannheim	153 [13.6%]	122 [11.4%]	2 [6.5%]	277 [12.4%]	105 [12.7%]	96 [12.5%]	0 [0.0%]	201 [12.5%]
Dresden	126 [11.2%]	139 [13.0%]	0 [0.0%]	265 [11.9%]	98 [11.9%]	112 [14.6%]	0 [0.0%]	210 [13.1%]

^a^Number of participants with unknown sex. For rows referring to age and BMI, frequencies correspond to number of participants with missing age/BMI data

^b^Relative or guardian that provided parent responses on the DAWBA

### Three-month prevalence of DEB

The prevalence of the seven DEBs was stratified by sex and informant (adolescent, parent or either informant [5]) at both time points (Table 2): prevalence was based on the total number of participants providing a presence/absence rating for each DEB. All DEBs were endorsed to a greater extent in adolescent reports compared to parent reports. Prevalences were highest when endorsement by either the parent or child was taken into account. However, there was only a small difference in prevalence estimates between adolescent reports and endorsements using either informant, whereas there was a notable difference in prevalence between either informant and parent reports. Similar prevalences were reported at both time points across informants. Of note, the prevalence of binge eating and purging almost doubled from age 14 to age 16 from adolescent reports, whereas the prevalence of binge eating decreased over time in parent reports. Across all informant types and DEBs, there was greater endorsement in girls compared to boys. Concordance was driven by agreement on the absence of the behaviour. Kappa estimates were between 0.045 and 0.318, suggesting at most slight to fair agreement. Given the large prevalence index (i.e. the difference between the number of dyads agreeing on the presence compared to the absence of the behaviour), the proportion of chance agreement is expected to be high, which may have contributed to the low kappa estimates [25].

**Table 2:** Prevalence of disordered eating behaviours according to adolescent report, parent report, or either informant report (using “OR rule” method), and percentage agreement

	Adolescent report^a^	Parent report^a^	Either informant^a^	Agreement^b^	
	% (% girls^c^) [*N* reporting symptom/*N* measured]	% (% girls^c^) [*N* reporting symptom/*N* measured]	% (% girls^c^) [*N* reporting symptom/*N* measured]	% (*N* pairs agreed [Agreed present/total *N*)]	Kappa statistic (95% confidence interval)
Age 14					
Fear of weight gain	39.6% (70.9%) [856/2159]	21.5% (71.4%) [465/2160]	45.3% (69.4%) [989/2185]	69.70% [337/2156]	0.316 (0.277, 0.355)
Distress over weight/shape	17.9% (77.2%) [386/2159]	6.5% (72.1%) [140/2161]	20.6% (74.7%) [451/2186]	82.80% [77/2157]	0.219 (0.168, 0.270)
Avoidance of fattening foods	28.8% (69.6%) [622/2159]	11.9% (73.9%) [257/2160]	33.0% (69.6%) [721/2185]	73.90% [161/2156]	0.236 (0.194, 0.278)
Food Restriction	29.2% (72.7%) [631/2158]	14.2% (75.9%) [307/2160]	34.4% (72.4%) [751/2185]	74.10% [190/2155]	0.265 (0.222, 0.308)
Exercise for weight loss	30.1% (67.1%) [650/2158]	10.2% (69.7%) [221/2160]	33.0% (66.4%) [721/2185]	73.55% [152/2155]	0.230 (0.190, 0.270)
Binge eating	4.9% (88.7%) [106/2159]	1.2% (72.0%) [25/2160]	5.7% (84.7%) [124/2185]	94.60% [7/2156]	0.090 (0.018, 0.162)
Purging	5.7% (77.4%) [124/2159]	0.6% (76.9%) [13/2161]	5.9% (76.0%) [129/2186]	94.40% [9/2157]	0.119 (0.046, 0.193)
Any symptom	43.4% (58.3%) [938/2159]	23.0% (31.6%) [498/2161]	48.9% (64.1%) [1070/2186]	67.55% [371/2157]	0.304 (0.267, 0.342)
Age 16					
Fear of weight gain	36.4% (78.0%) [568/1560]	16.7% (77.5%) [258/1543]	39.9% (76.4%) [635/1591]	72.00% [193/1524]	0.318 (0.271, 0.364)
Distress over weight/shape	21.7% (81.7%) [339/1560]	5.3% (80.5%) [82/1543]	23.3% (80.5%) [370/1591]	80.05% [52/1524]	0.186 (0.132, 0.239)
Avoidance of fattening foods	26.8% (78.0%) [418/1560]	10.6% (78.7%) [164/1543]	30.0% (77.4%) [477/1591]	76.60% [107/1524]	0.262 (0.210, 0.314)
Food Restriction	31.0% (80.6%) [484/1560]	12.5% (77.2%) [193/1543]	34.2% (79.0%) [544/1591]	74.30% [135/1524]	0.278 (0.229, 0.328)
Exercise for weight loss	27.0% (75.8%) [421/1560]	9.9% (73.2%) [153/1543]	29.9% (74.4%) [476/1591]	76.18% [101/1524]	0.247 (0.196, 0.299)
Binge eating	8.6% (85.1%) [134/1560]	0.6% (70.0%) [10/1543]	8.8% (84.3%) [140/1591]	91.30% [4/1524]	0.045 (-0.005, 0.095)
Purging	10.4% (79.8%) [163/1560]	1.4% (76.2%) [21/1543]	10.7% (78.9%) [171/1591]	89.70% [13/1524]	0.121 (0.056, 0.186)
Any symptom	40.7% (57.9%) [635/1560]	17.7% (26.0%) [273/1543]	44.0% (61.9%) [700/1591]	69.23% [211/1524]	0.304 0.299 (0.255, 0.343)

^a^Only included individuals with adolescent sex reported

^b^Included all individuals (regardless of whether sex was reported/missing)

^c^% of the sample reporting the symptom who were girls

### Stability of symptoms and changes in informant reports

To explore whether concordance improved over time if the symptoms persisted, parental reports were assessed specifically for adolescents who reported the presence of any symptom at both time points and vice versa (Table 3).

**Table 3:** Informant agreement at T1 and T2 for dyads in which symptoms were endorsed by one informant at both time points

Of the dyads in which:	Adolescents endorsing symptoms at both time points				Parents endorsing symptoms at both time points				Number of parents and adolescents who endorsed symptoms at both ages*
Symptom	Number of adolescents reporting symptom at both time points	Number of adolescents for whom one parent rating was missing	Number of adolescents for whom symptoms were endorsed by parents at age 14	Number of adolescents for whom symptoms were endorsed by parents at age 16	Number of parents reporting symptom at both time points	Number of adolescents for whom one adolescent rating was missing	Number of parents who endorsed symptoms at age 14	Number of parents who endorsed symptoms at age 16	
Fear of weight gain	409	18	172	150	159	1	121	120	103
Distress over weight/shape	156	5	41	35	38	0	29	27	21
Avoidance of fattening foods	267	10	82	77	69	2	49	48	40
Food restriction	295	14	91	93	89	1	61	61	49
Exercise for weight loss	261	10	69	80	64	1	49	43	39
Binge eating	32	1	2	2	3	0	1	1	0
Purging	49	0	1	4	1	0	1	1	1
Any symptom	461	20	194	172	171	2	136	134	120

Note: Participants with missing sex information were included in this analysis

* These individuals were included in the separate frequency counts at ages 14 and 16 years

In a combined assessment of bulimic behaviours, 83 adolescents endorsed binge eating and/or purging behaviours at both time points, whereas only 5 parents reported these behaviours at both time points. For only 3 of these parent–adolescent dyads, at least one of these behaviours was reported at both time points by both informants. Of the adolescents who endorsed bulimic behaviour(s) at both time points, 4 parents reported the behaviour at age 14 [binge eating (*n* = 3), binge eating and purging (*n* = 1)], whereas at age 16, the behaviours were reported by 8 parents [binge eating (*n* = 2), purging (*n* = 5), binge eating and purging (*n* = 1)]. Of the parents who reported least one of these behaviours at both time points, all adolescents reported the behaviour at one or more time points: 1 reported the bingeing at T1 only, 1 reported purging at T2 only, and 3 reported binge eating/purging at both time points (2 reported both behaviours, 1 reported binge eating at T1 and purging at T2).

### Modelling prevalence based on informant and the adolescent’s sex and age

Table 4 presents the estimated prevalence of each symptom predicted by the informant, the adolescent’s sex and the adolescent’s age. The odds for the behaviour being endorsed by girls compared to boys, adolescents compared to parents, and at age 14 compared to 16 were determined by calculating the exponentiated beta coefficient (odds ratio; OR).

**Table 4:** Modelling prevalence by adolescent’s age, adolescent’s sex and informant: Odd’s ratios (OR) [95% confidence intervals (CI)] calculated using each sex, informant and time point as the reference category

Symptom	Sex			Informant			Age		
(Reference category)	(Girls)	(Boys)	*p*	(Adolescent)	(Parent)	*p*	(T1)	(T2)	*p*
Fear of weight gain									
OR (95% CI)	0.241 (.209, .279)	4.145 (3.585, 4.793)	<0.001	0.369 (.331, .411)	2.711 (2.435, 3.017)	<0.001	0.744 (.674, .821)	1.344 (1.217, 1.484)	<0.001
Distress over weight/shape									
OR (95% CI)	0.230 (.189, .280)	4.344 (3.569, 5.287)	<0.001	0.252 (.216, .295)	3.963 (4.636, 3.388)	<0.001	1.057 (.930, 1.202)	0.946 (.832, 1.075)	0.393
Avoidance of fattening foods									
OR (95% CI)	0.285 (.242, .334)	3.514 (2.990, 4.131)	<0.001	0.316 (.280, .356)	3.169 (2.810, 3.573)	<0.001	0.823 (.738, .918)	1.214 (1.090, 1.354)	<0.001
Food restriction									
OR (95% CI)	0.232 (.198, .273)	4.306 (3.669, 5.054)	<0.001	0.344 (.306, .387)	2.907 (2.583, 3.272)	<0.001	0.934 (.837, 1.042)	1.071 (.960, 1.945)	<0.001
Exercise for weight loss									
OR (95% CI)	0.338 (.289, .395)	2.962 (2.533, 3.464)	<0.001	0.269 (.237, .304)	3.722 (3.284, 4.217)	<0.001	0.833 (.746, .930)	1.200 (1.075, 1.340)	0.001
Binge eating									
OR (95% CI)	0.169 (.117, .246)	5.901 (4.067, 8.561)	<0.001	0.134 (.092, .196)	7.440 (5.110, 10.832)	<0.001	1.469 (1.166, 1.850)	0.681 (.541, .857)	0.001
Purging									
OR (95% CI)	0.273 (.202, .369)	3.667 (2.712.957)	<0.001	0.111 (.078, .160)	9.049 (6.371, 12.851)	<0.001	1.876 (1.508, 2.332)	0.533 (0.663, 0.429)	<0.001
Any symptom									
OR (95% CI)	0.272 (.235, .315)	3.680 (3.178, 4.261)	<0.001	0.330 (.298, .365)	3.030 (2.738, 3.352)	<0.001	0.831 (.906, .762)	1.204 (1.104, 1.313)	<0.001

Estimated marginal means (EM) were converted into percentages to reflect the prevalence of the behaviour according to predictor variable. Coefficients were exponentiated to present odds ratios (OR). All models were run on imputed datasets. The parameters presented are pooled estimates combined using Rubin’s rules

Girls were 2.96–5.90 times more likely to endorse any of the DEBs than boys. With the exception of distress over weight/shape, the differences in predicted prevalence of DEBs between age 14 and 16 were small yet statistically significant, though the direction of the differences was inconsistent. The prevalence of fear of weight gain, avoidance of fattening foods, food restriction and exercise for weight loss were 1.07–1.34 times higher at age 14 than at age 16, whereas binge eating and purging were 1.47–1.88 times more prevalent at age 16 than 14. Finally, adolescents were at least 2.71 times more likely to report any DEB than their parent. This was especially true for bulimic behaviours: adolescents were 7.44 times more likely to report binge eating and 9.05 times more likely to endorse purging compared to parent reports.

## Discussion

This study assessed the three-month prevalence of DEB across mid-adolescence in a multinational European sample based on adolescent and parent reports, and investigated the degree of concordance between informants. It was predicted that DEBs would be more prevalent amongst girls compared to boys, adolescent reports compared to parents, and at age 16 compared to age 14.

Our data revealed that DEBs were more consistently endorsed amongst girls compared to boys and adolescents compared to their parents. However, prevalence did not vary widely as a function of age. This may be due to similarities between ages 14 and 16 years in terms of physical development (i.e. post-pubertal) and environmental challenges. Greater differences in prevalence may be expected if comparisons are made between time points characterised by different environmental challenges and both physical and neural developmental stages, i.e. pre- versus post-puberty, or early versus late adolescence. For example, in the context of clinical diagnoses, Allen et al. [26] reported that the prevalence of EDs increased significantly from age 14 to ages 17 and 20 in females but only between ages 14 and 20 in males. Thus, future studies may wish to model the impact of the interaction between age and sex on symptom prevalence.

DEBs were more prevalent amongst girls compared to boys at both time points. Although the magnitude of the sex differences observed in this study is smaller than that reported in treatment-seeking samples, it is of similar magnitude to population-based studies in adolescents [5, 17, 27, 28]. However, there is some inconsistency regarding the direction and specificity of the differences in prevalences between sexes [29]. For example, one recent study in the UK found a greater prevalence of some DEBs in girls compared to boys using parental reports (including fear of weight gain, distress about weight/shape, food restriction and avoidance of fattening foods), but equivalent endorsement of binge eating and purging across the sexes (approx. 5 and 0.2%, respectively) [17]. Another study reported a greater prevalence of binge eating amongst girls compared to boys in adolescent self-reports, but no sex-related differences in parent reports [5]. In contrast, we found a greater prevalence of all DEBs in girls (including binge eating and purging) in both the parent and adolescent reports, suggesting that the sex-related differences in prevalence are not simply a matter of the informant. However, factors such as issues in general recognition of DEBs in boys or willingness to report may have contributed to these findings. For example, Lee-Winn et al. [30] reported that although no sex differences were observed with respect to recurrent overeating, emotional aspects of binge eating (loss of control, distress) were more prevalent in girls than boys. These authors suggested that this may be due to emotional expression being seen as less socially acceptable amongst boys.

Consistent with previous studies, our data revealed slight to fair concordance between parent and adolescent reports of ED symptoms. All symptoms were more prevalent amongst adolescent self-reports compared to parent reports, particularly with respect to binge eating and purging. This may be due to the secrecy and shame often associated with these behaviours (Wilfley et al. [31]). We also found that the number of parents reporting binge eating and/or purging in adolescents who report themselves as symptomatic at both time points does not improve over time. However, contrary to Swanson et al.’s [5] findings of increased prevalence estimates for some DEBs (e.g. binge eating, fasting) DEBs obtained using the “OR” rule (i.e. reported by either informant) compared to reports from adolescents/children only, combining parent and adolescent reports in our study elicited prevalence estimates that were only slightly higher than those from the adolescent reports alone for all DEBs. In contrast, they were substantially higher than the prevalence estimates from parent reports. These findings suggest that parents are unaware of their children’s endorsement of such behaviours [15]. The lack of awareness of adolescents’ binge eating and purging in mid-adolescence may contribute to a central issue in EDs, namely difficulty in early recognition. If this is correct, it is important to educate parents, raise awareness of these symptoms and to take note of symptoms in late childhood and early adolescence. However, such discrepancies may also be explained by differences in attitudes towards, understanding or interpretation of DEBs [4, 5]. Future studies may wish to investigate the similarity of interpretations of behavioural definitions between informants to address issues of comparability between reports.

The main strength of this study is the use of a large, representative, multinational community sample with repeated measurements at 2 time points and a range of parental/caregiver figures. We considered a range of ED symptoms and how the persistence of symptoms over 2 years may influence adolescent–parent concordance. Although we did not take into account the contact time between parents and adolescents, which may influence the parent’s awareness of the adolescent’s behaviour, it is expected that our sample encapsulates a broad array of parental involvement. Moreover, the use of a community sample may explain the high rates of concordance for the absence of DEBs [13].

This study has some limitations. Firstly, due to the use of skip rules in the DAWBA, the majority of the “absent” responses were presumed absent based on negative responses to all five screening questions. Thus, individuals who may engage in these behaviours although they did not endorse the screening questions would not have been accurately represented. However, post hoc exploration of the endorsement of entry questions yielded similar findings to our GEE models, whereby entry questions were more frequently endorsed in girls compared to boys (by both informants) and by adolescents compared to parents (for both sexes), and this did not vary widely between time points: (adolescents: 58.0–58.5% girls and 23.1–29.7% boys; parents: 20.9–25.2% girls and 5.3–9.6% boys). Secondly, there were insufficient data to investigate differences in symptom severity. Finally, parental weight and parental history of an ED were not assessed. Parents experiencing difficulties with eating or weight regulation may not be as good at noticing eating problems in their children [32]. Moreover, maternal and paternal BMI have been reported to be predictors of ED caseness at age 14 compared to healthy controls and psychiatric controls, respectively [33]. Therefore, parental weight and disordered eating should be included as predictor variables in future models of symptom prevalence.

Our findings suggest that adolescent reports (compared to parent reports) elicit greater prevalence of ED symptoms in mid-adolescence in the community. It is unclear, however, whether the behaviours are being over- or under-reported by adolescents and parents, respectively. While the use of multiple informants can provide a broader picture of the adolescent’s behaviour, they may also introduce errors with inaccurate reporting of behaviour. Given the limitations of self-report, alternative perspectives provided by a second informant may be useful in identifying potentially vulnerable individuals who may not identify themselves as symptomatic, or for conceptualising an adolescent’s behaviour in different contexts. Indeed, there has been some support for the use of computerised diagnostic assessments such as the DAWBA that takes into consideration multiple informants’ reports to calculate the probability of a child/adolescent having an ED [34, 35]. However, a Swiss study of child and adolescent outpatients comparing the ICD-10 diagnoses provided by clinicians to those reached through expert review of DAWBA data revealed that although agreement between the DAWBA expert and clinician ratings was observed, this was largely driven by negative ratings of EDs [36].

In the context of diagnoses determined using information from multiple informants, the question remains as to how to prioritise or combine this information. While parent reports may be superior and more practical when assessing young children [37], it is likely that adolescents will be sufficiently aware of their own thoughts/behaviours, and thus may be a reliable source of information. Additionally, parent reports may be useful in determining symptom severity, which this study was unable to assess. For example, it has been suggested that persistent disagreement between informants could indicate a poorer prognosis compared to individuals for whom informant ratings consistently converge [38]. However, parental reports may be important with respect to the denial that often occurs at the start of the illness, particularly in AN [6]. Indeed, in the present study, 51 additional cases were identified by parental reports as endorsing any DEB at both time points (who were not also endorsed at both time points by adolescent reports); however, this is substantially smaller than the 341 additional cases identified by adolescent reports. To better understand the utility of multiple informants in identification of DEB in adolescents, future studies should aim to clarify how concordance rates differ throughout childhood and adolescence in relation to a broader array of symptoms, the factors that influence concordance (e.g. parental weight/eating difficulties) and how this relates to prognosis and the duration of untreated symptoms/ED.
